# Identifying preferred format and source of exercise information in persons with multiple sclerosis that can be delivered by health‐care providers

**DOI:** 10.1111/hex.12541

**Published:** 2017-05-18

**Authors:** Yvonne C. Learmonth, Brynn C. Adamson, Julia M. Balto, Chung‐yi Chiu, Isabel M. Molina‐Guzman, Marcia Finlayson, Barry J. Riskin, Robert W. Motl

**Affiliations:** ^1^ School of Psychology and Exercise Science Murdoch University Murdoch WA Australia; ^2^ Department of Kinesiology and Community Health University of Illinois at Urbana‐Champaign Urbana IL USA; ^3^ Department of Latina/Latino Studies University of Illinois at Urbana‐Champaign Urbana IL USA; ^4^ School of Rehabilitation Therapy Queen's University Kingston ON Canada; ^5^ Department of Physical Therapy University of Alabama at Birmingham Birmingham AL USA

**Keywords:** exercise promotion, health‐care communication, multiple sclerosis, qualitative

## Abstract

**Background:**

There is increasing recognition of the benefits of exercise in individuals with multiple sclerosis (MS), yet the MS population does not engage in sufficient amounts of exercise to accrue health benefits. There has been little qualitative inquiry to establish the preferred format and source for receiving exercise information from health‐care providers among persons with MS.

**Objective:**

We sought to identify the desired and preferred format and source of exercise information for persons with MS that can be delivered through health‐care providers.

**Setting and participants:**

Participants were adults with MS who had mild or moderate disability and participated in a range of exercise levels. All participants lived in the Midwest of the United States.

**Methods:**

Fifty semi‐structured interviews were conducted and analysed using thematic analysis.

**Results:**

Two themes emerged, (i) approach for receiving exercise promotion and (ii) ideal person for promoting exercise. Persons with MS want to receive exercise information through in‐person consultations with health‐care providers, print media and electronic media. Persons with MS want to receive exercise promotion from health‐care providers with expertise in MS (ie neurologists) and with expertise in exercise (eg physical therapists).

**Conclusions:**

These data support the importance of understanding how to provide exercise information to persons with MS and identifying that health‐care providers including neurologists and physical therapists should be involved in exercise promotion.

## 1 INTRODUCTION

Multiple sclerosis (MS) is a disabling, degenerative and chronic neurological disease of the central nervous system (CNS).^1^ Worldwide, the prevalence of MS is thought to be increasing with upwards of 2.5 million people living with the disease.^2^ The damage within the CNS manifests as an increase in fatigue, motor weakness, heat sensitivity, reduced mobility, abnormal gait biomechanics, altered balance, cognitive deficits and autonomic dysfunction. It has previously been stated that rehabilitation [is] still the only way to improve function in MS,^3^ and for 2 decades, there has been increasing focus on identifying rehabilitation strategies to manage such consequences in persons with MS.^4^, ^5^, ^6^, ^7^

Exercise is one rehabilitation strategy that has substantial evidence of efficacy in the management of many common symptoms of MS.^8^, ^9^, ^10^, ^11^ Researchers have suggested that exercise training may be the single most effective non‐pharmacological approach for managing symptoms,^12^, ^13^ and improving health‐related quality of life in persons with MS.^14^ However, a significant number of persons with MS are not engaging in sufficient exercise to accrue health benefits.^15^, ^16^

A consensus meeting titled *Exercise as a Prescriptive Therapy in Multiple Sclerosis* provides a strong statement for exercise as one of the best therapies available for inclusion in the comprehensive care of patients with MS.^12^ The chronic degenerative nature of MS results in lifelong interactions between patients and health‐care providers, and these patient‐provider interactions may be critical for exercise adoption and maintenance. There is evidence that persons with MS expect exercise promotion from health‐care providers,^17^, ^18^, ^19^, ^20^ but these patients might not currently be receiving it.^17^, ^21^, ^22^ We recently provided new information that identified the needs and wants of patients regarding exercise promotion by health‐care providers through a qualitative research study of 50 persons with mild‐to‐moderate MS.^17^ The qualitative data supported previous findings 19, 20, 22 and more clearly indicated that persons with MS need and expect health‐care providers to promote and provide information on exercise promotion. Our evidence is in line with general communication deficits between patients and providers in MS healthcare globally^23^ and highlights the urgency of understanding and developing structured exercise communication between patients with MS and health‐care providers as part of the patient provider interaction.^24^, ^25^

The next step in this line of research involves identifying the format and source of exercise information that is wanted by persons with MS. Indeed, our previous qualitative study was comprehensively designed a priori for addressing this next research step, and we approached it separately in this study given the breadth and depth of data collected in the qualitative research we undertook. The current study will identify the preferred format through which persons with MS would like to receive exercise promotion information from health‐care providers and the preferred source to receive exercise promotion information. This information will provide practical evidence on the optimal format and source of exercise promotion information delivered within the health‐care context.

## 2 METHOD

The current research represents a further presentation of data collected during a qualitative research study that identified the health‐care experiences of persons with MS regarding exercise promotion.^17^ The current research was a priori defined so that it extended the initial publication from a large, qualitative research undertaking. We received ethical approval from a university institutional review board and written consent from all research participants. All data were anonymised prior to transcription. We adopted a participatory framework^26^ and included important aspects of patient and public involvement and engagement (PPIE)^27^ in the design, conduct, analysis and dissemination plans of this study. We invited persons with MS and health‐care providers known to the research group to collaborate on the study. We conducted research scoping meetings with our collaborators, and this helped us establish the design and methodology; for example we discussed gathering data via interviews, the content of interviews and participant inclusion criteria. Our collaborators who were experienced qualitative researchers further advised and assisted on the analysis of the data, and all collaborators advised and agreed upon the dissemination of results.

We used interpretative descriptive methodology (IDM),^28^ and this allowed us to examine the person's life‐experiences and opinions relevant to the clinical context of applied rehabilitation^29^ and has been developed to ease the interpretation of patterns and themes emerging from clinical phenomena.^30^ IDM further acknowledges that the presentation of results is guided by the authors’ professional and personal opinions and knowledge. IDM is well suited for establishing the preferred mechanisms through which persons with MS would like to receive exercise promotion information from health‐care providers, as it has been used in many past studies to analyse the life‐experiences of those living with MS.^17^, ^24^, ^31^, ^32^, ^33^

To add credibility to our research, we incorporated participant reflection into our methodology^34^ by inviting participants to provide further comment post‐interview, and this was achieved through the use of (i) participant take‐home journals which contained prompts of the main questions discussed, and (ii) a one‐page interview summary sheet written by interviewers immediately post‐interview. Both items were provided to participants to encourage reflection after the interview and served to (i) provide further data and (ii) allow researchers to establish whether post‐interview opinions were similar to those expressed during the interview.

### 2.1 Participant recruitment

**Figure 1:**
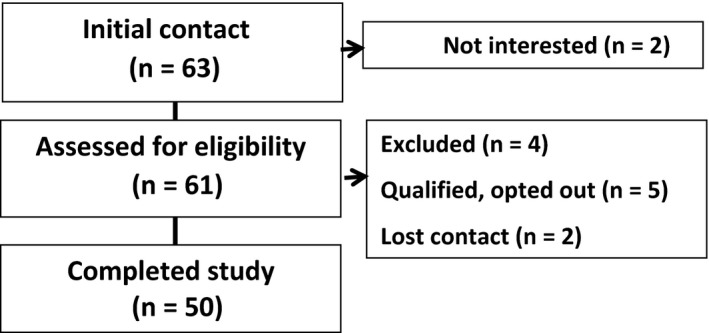
Participant recruitment

Participants were recruited from the Midwest of the United States. To reach a large number of potential participants and reduce bias to any one form of information seeking, we chose two diverse recruitment strategies. We informed persons with MS about the study through (i) online advertisement on the National MS Society and University of Illinois websites and (ii) presentations by our research staff at regional National Multiple Sclerosis Society meetings and events. Sixty‐one persons with MS were screened for eligibility: (i) age over 18 years; (ii) physician‐confirmed verification of MS diagnosis; (iii) no MS relapse within past 30 days; (iv) self‐report Expanded Disability Status Scale (SR‐EDSS) score ≤5.5; and (v) willingness to be audio‐recorded during interviews. Recruitment and reasons for non‐inclusion are listed in Figure 1; 50 persons were interviewed once by one of three researchers in a private room within our research site, and interviews lasted approximately 45 minutes. Ten participants (20%) were known to researchers prior to the study, based on participation in previous research studies, but we did not target such persons for participation; this further was not an exclusion criteria. Our analysis continued concurrently with our interviews, and recruitment ceased after 50 interviews as at this point no new themes were occurring (ie saturation).

### 2.2 Sample diversity

We aimed to capture experiences and opinions from persons with MS who had a range of activity levels who all had mild‐to‐moderate disability as a result of MS. We confirmed disability status during a neurological examination using the Expanded Disability Status Scale (EDSS).^35^ During the screening phone call, we established current activity level (insufficiently active or sufficiently active) using the Godin Leisure‐Time Exercise Questionnaire (GLTEQ),^36^ where scores of ≤27 represented insufficiently active and scores of ≥28 represented sufficiently active according to public health guidelines.

### 2.3 Procedure

Data were collected through semi‐structured interviews. Interview content validity was confirmed via our inclusion of topics that emerged from formative and purposeful interactions with persons with MS (ie PPIE),^37^ the literature and discussions among the research team that included patients with MS and MS health‐care providers. Interviewers had more than 4 years of experience conducting qualitative research in MS, and we ensured between‐interview reliability via the use of the same standardised basic interview outline and prompts in all interviews.

We asked all participants about receiving exercise promotion information from health‐care providers. The interviews began with general questions such as *“Let's talk about your experience with exercise?”* This question was then followed by more exploratory questions such as “*Where do you look for information on exercise; would that be a good format to receive exercise guidance?”, “What type of information or guidance would you want from your healthcare provider to help you exercise?”,* and *“Ideally, what healthcare provider would you like to discuss exercise with?”* We conducted the interviews to contain as much in‐depth information as possible, and researchers were free to use inductive reasoning throughout the interview to ensure that rich data were generated.

We administered a standardised survey to capture background information on the participants’ demographical (ie age, sex) and clinical (ie type of MS and years since diagnosis [YSD]) characteristics. Participants received a journal containing the main interview questions, and a summary sheet typed by interviewers immediately post‐interview; this was for the purpose of collecting further reflections. We requested that participants return the journal and summary sheet for making further comments, and we provided a pre‐stamped, pre‐addressed envelope. The reflective journals were analysed alongside the transcriptions. No participants returned the summary sheet.

### 2.4 Analysis and presentation

Our analytic method was discussed and approved by all researchers which included persons with MS. The interviews were audiotaped, transcribed and then analysed using IDM.^28^ Participants’ comments from returned journals were added into respective interviews in relevant locations within the interview transcription. Researchers then listened to the interviews and read the transcripts. We analysed and organised our data following spiral analysis,^38^ and this technique complements IDM as it encourages repeated immersion in the data. Our technique included listening to the interviews and organizing the data; reading and memoing the data; describing, classifying and interpreting data into codes and themes; and finally representing and visualizing the data. Throughout the process, we kept researcher Reflective Analysis Notes and we asked ourselves “What are the main thoughts we are learning from this interview?” “Why is this participant saying that?” “How does this compare with the literate?” “How does this compare with other interviews?” “What do the thoughts in this interview mean to the grander scheme?” “How does the participants interview thoughts compare with what we know about the participant?” We frequently returned to, and developed, our analytical questioning of the interview analysis as we attempted to interpret the interview, and this process provided a coherent analytical framework of interpretative description.^39^ Participant demographical information (ie MS subtype, sex, age, disability level, exercise level and years since diagnosis [YSD]) were considered during analysis of each interview and later analysis of the entire sample. Analysis was performed by three researchers (BCA, JMB and YCL). We used inductive analysis to create a coding book, and this was based on open coding of six randomly selected interviews; open coding was performed independently by each researcher, and then, we met to finalise the code book. Codes were unique ideas presented by a group of participants, and within our codebook, we provided descriptors that guided researcher coding (eg Paper—participant indicates they would like a hard copy of the information), and subthemes were created from codes we considered similar (eg Print media—paper handouts and pamphlets). We engaged with our wider research team to discuss our findings and made appropriate modifications to our analysis. BCA, JMB and YCL then independently coded the remaining interviews, and all interviews were coded by two researchers who then met to finalize analysis. All three researchers discussed further modifications to the coding book, as appropriate. Finally BCA, JMB and YCL compiled the main themes emerging from the data, and these themes were discussed with the wider research team and disseminated to patients, health‐care providers and researchers during a national MS conference presentation^40^ and on our previous laboratory website, and regional support group meetings.

### 2.5 Quality and credibility

We included patients and health‐care providers in the design, analysis and dissemination plan for our research. We listened to and acted upon comments from participants in our previous research studies of patient with MS^37^, ^41^ when designing and conducting our study. We invited persons with MS and clinicians to participate in our research as advisors and co‐authors. We considered feedback from the wider team and persons attending a national MS conference in the final manuscript presentation (eg clinicians encouraged us to provide depth of data on the patients perceived ideal person to promote exercise).

We ensured credibility and dependability through triangulation in our analysis wherein our primary research team independently and jointly analysed interviews and had frequent discussions with our wider research team. We ensured consistency within our primary research team by undertaking pilot interviews with persons with MS before beginning the study. We further used semi‐structured interview scripts and weekly meetings to discuss interviews, transcripts and analyses.^42^ We further increased credibility in our qualitative data analysis by creating a coding book early in our analysis, and this code book was refined throughout our analysis. We applied similar codes in all interviews, and all researchers used the same codes. Triangulation of sources was achieved through analysis of the transcribed interview and comments from participants’ journals.^38^, ^42^

To facilitate our research findings being available to the widest possible audience (eg patients with MS and health‐care providers),^43^ we considered comments from patients with MS who had previously told us about valuing open‐access research findings, and we acknowledged the importance patients with MS place in accessing information on the Internet.^44^ To that end, our wider research team agreed to the dissemination of results in an open‐access health‐care journal.

## 3 RESULTS

Data were analysed from all 50 interviews. All participants completed the survey providing demographic and clinical information, and these are presented alongside data on the overall disability level (ie EDSS) and current exercise level (ie GLTEQ) of the sample in Table 1.

**Table 1:** Demographics of sample (n=50)

Age	49.2 (10.3)
Sex F/M	33/17
EDSS^a^	3.5 (2)
GLTEQ	24.1 (16.7)
Time since diagnosis	13.0 (8.4)
MS Type (RR/SP/PP/B)	41/5/1/3

EDSS, Expanded Disability Status Score; GLTEQ, Godin Leisure‐Time Exercise Questionnaire RR, Relapsing‐remitting; SP, Secondary Progressive; PP, Primary Progressive; B, Benign.

Means and SDs are reported unless otherwise indicated.

a Median (Interquartile Range).

### 3.1 Demographic characteristics

The mean age of the sample was 49.2 (SD: 10.3, median: 51.5) years. The majority of participants were female (n=33). Clinically, participants had been diagnosed with MS for a mean of 13.0 (SD: 8.4) years, and typically had relapsing‐remitting MS (n=41) with a median EDSS score (3.5, IQR: 2) that indicated mild‐to‐moderate neurological disability. Regarding current exercise behaviour, the mean GLTEQ score was 24.1 (SD: 16.7). Of note, we interviewed 31 participants with mild MS (median EDSS=3, IQR: 1.5), and 19 participants with moderate MS (median EDSS 4.5, IQR: 2.1). We interviewed 31 participants who were insufficiently active (mean GLTEQ 15.7, SD: 8.7), and 19 participants who were sufficiently active (mean GLTEQ 38.5 [SD: 16.7]).

### 3.2 Thematic findings

We analysed data from all 50 interviews. Eighteen participants returned journals comments that were added to the typed interview script and analysed accordingly. We present results from two main themes: (i) Approach for receiving exercise promotion (ie what is the optimal format of exercise promotion information?) and (ii) Ideal person for promoting exercise (ie who is the optimal source of exercise promotion information?). Our results represent the preferred format and source of exercise information for the entire sample; that is, we did not identify any formats or sources unique to participants based on MS subtype, sex, disability level, exercise level or years diagnosed.

#### 3.2.1 Theme 1: Approach for receiving exercise promotion

This theme characterised the different formats through which patients with MS want to receive information related to exercise promotion. Participants indicated different formats for receiving the exercise promotion information, and this formed three subthemes: *traditional in‐person patient‐provider clinical consultation, print media* and *electronic media,* and these are displayed in Table 2.

**Table 2:** Overall findings

Theme	Subtheme
Approach for receiving exercise promotion.	Traditional in‐person patient‐provider clinical consultation Print media: Paper handouts and pamphlets Electronic media: DVD, on‐line and email
Ideal health‐care provider for promoting exercise	MS expertise Exercise expertise

##### 3.2.1.1 Traditional in‐person patient‐provider clinical consultation

Almost two‐thirds of participants discussed a preference for receiving exercise promotion information in‐person, directly from a health‐care provider. The participants preferred receiving the information within the health‐care provider's clinic, and less commonly in the health‐care provider's gym (eg physical therapist's gym) or the patient's home.

All participants discussed the importance of having questions answered and concerns addressed during the interaction with the health‐care provider. Participants further commented that the interaction with the provider should provide detailed information such as the format of an exercise programme, or a referral to another expert professional.

Verbal conversation's good, because usually that's going to back or reiterate whatever information that you've got or found via computer, via magazine. Wherever you find it when you hear it from somebody else that reinstates it or brings it home a little bit more, to reinforce it. It could help make a difference. Participant 12. Relapsing‐remitting (RR) MS, female (F) ≤54 years of age, moderate disability, insufficiently active, YSD>3 years

These preferences were wanted by persons with MS despite their current activity level or disability level, and this might reflect that many persons with MS currently do attend clinical consultations where exercise promotion opportunities should be provided. By contrast, we previously identified 17 that few persons with MS have recent experience with a physical therapist, or gym environment and as such may not envision this as an option without the suggestion or experience.

Participants further acknowledge that in‐person patient‐provider clinical consultations would allow for the correction of exercise movements and prevent incorrect form or potential injuries. Many participants considered the importance of being motivated to exercise by the commitment of attending a clinical appointment. We consider this an aspect of external accountability and identified that external accountability was wanted by all participants to a lesser or greater degree. The example from the female participant below reflects a need for a high level of external accountability.

A specific schedule. I have to be there, committed to being there. And again, someone that's doing it with me, and watching. I guess I need that stimulation of someone there with me. Participant 50, RR MS, F, ≤54 years of age, moderate disability, sufficiently active, YSD>3 years.

##### 3.2.1.2 Print media: Paper handouts and pamphlets

Over three‐quarters of participants discussed wanting to receive information written on print media (ie pamphlets, leaflets or instruction booklets). The main reason was that it acted as a memory aid and provided information that was easily accessible; *“I have reference to go because if I get busy then I'm like what was I supposed to do or how much is that. I can go check the book.”* Participant 014 (RR MS, F, >55 years of age, insufficiently active, TSD>3 years). Cognitive deficits are common in person with MS, and the high number of participants in this study requesting a “memory aid” for exercise promotion may reflect their awareness of cognitive deficits. We consider this a positive finding in that persons with MS are aware of personal limitations and vocalise their need for solutions to these deficits. Participants suggested different formats that would be helpful and flexible to meet their needs, and these included written and illustrated information which was clear and simple to use.

I think if you had a little flip chart with exercises, maybe things that were interesting to people. Participant 30, RR MS, male (M), ≤54 years of age, mild disability, insufficiently active, TSD>3 years.

I guess I would say something … some brochure, something like that simple, to the point, positive type thing. You get too much and people are like, ‘ehh, too much’. Participant 28. RR MS, F, ≤54 years of age, mild disability, insufficiently active, YSD>3 years.

Occasionally, some participants told us that receiving information in print format may not be ideal. This was either because print media information was associated with a lot of reading that was perceived to be difficult, or because printed information could be easily lost or misplaced. Participants deemed exercise promotion material as important and did not want to lose it, and this reflects the value they place on exercise information.

##### 3.2.1.3 Electronic media: DVD, online and email

Almost half of participants were interested in receiving exercise promotion via electronic media sources, including DVDs, websites with written, pictorial or video content, and email. Participants had experience following exercise DVDs, and many indicated this was helpful for following an exercise routine at home. Others wanted instructional videos on exercise promotion rather than solely an exercise programme. We consider that these suggestions may be based on participants current experiences and reflect that persons with MS will seek out familiar information sources unless suggested otherwise. Again, it was important for the information to be clear and simple to use.

I could see that perhaps for some people that would maybe be helpful if you had a yoga session on DVD that was designed for people with MS that they could just go home and do, that's something I've done before. Participant 27, RR MS, F, ≤54 years of age, mild disability, sufficiently active, YSD>3 years.

A video or DVD with some video on it so they are more talking to you instead of just you read through and you go from there Participant 35, secondary progressive (SP)MS, M, ≤ 54 years of age, moderate disability, insufficiently active, YSD>3 years.

Participants told us that information provided via electronic media should be up‐to‐date, interactive and offer greater depth of detail online and that it should be accessed from anywhere (eg at home and on smartphones). Some gave examples of health‐care providers using electronic communications successfully and suggested that this could be taken further with exercise promotion. For instance, participants wanted to receive direct emails on exercise opportunities in the local community or information about the latest research or exercise equipment.

They (participant healthcare provider) already have my email, for appointment reminders I think, so I would find that helpful if, then by location, I was emailed what's going on sports and exercise‐wise in my area. Participant 19, RR MS, F, ≤ 54 years, moderate disability, insufficiently active, YSD>3 years.

Some participants would like to communicate directly with a health‐care provider using electronic means. As discussed, participants indicated that email contact was good and acknowledged that video conferences with health‐care providers were a good way to add accountability without needing to attend traditional clinical appointments.

It would be nice to have the individual attention of a face‐to‐face encounter. You could do that online just as easily almost. And that would work for me. Participant 40, RRMS, F, ≤ 54 years of age, moderate disability, insufficiently active, YSD≤3 years.

Over one‐third of participants acknowledged problems and reasons for preferring a minimal amount of exercise promotion via electronic media. These examples were often contrived from negative personal experiences, and this might not mean that these formats are not suitable if the information was delivered in a clear and simple manner. In most cases, negative opinions were not related to receiving directed health‐care promotion. Most negative opinions were a reference to receiving an abundance of emails already and worrying about not prioritizing exercise promotion when received through email. Others complained that there was an overwhelming volume of information available on the Internet and an associated inability to identify what information was appropriate. “*If you say here's a website to go to, then that kind of gets lost among all the other websites, and I'll forget where I started.”* Participant 25 (RR MS, M, <55 years of age, sufficiently active, TSD>3years). Some participants admitted to not being confident using technology, and in these cases, participants voiced concern about not correctly following exercise information provided to them in an electronic format.

### 3.3 Theme 2: Ideal person for promoting exercise

This theme characterises who the ideal exercise promoter would be for persons with MS, within the context of the provider's professional expertise. Two subthemes were identified: *MS expertise* and *exercise expertise,* and these are presented in Table 2.

#### 3.3.1 MS expertise

Over half of participants would prefer exercise promotion from a source knowledgeable in MS. This was because participants deemed that such a source would have a good understanding of disease progression and symptoms experienced as a result of MS and that these areas would be prioritised when promoting exercise.

Because he[my neurologist] really… knows MS and anything that's going on with me, he always has a suggestion or a recommendation. So he's got a wealth of knowledge and pretty much knows what could happen, predicts, and things like that, long before I could, so he would be the person. Participant 31. RR MS, F, > 55 years of age, mild disability, insufficiently active, YSD>3 years.,

In many cases, the participant's choice was based on previous positive experiences with a health‐care provider (ie neurologist). If participants perceived that a neurologist was knowledgeable in topics such as exercise, the participants were more likely to deem neurologists as the ideal source of exercise information.

From the effects of MS yes, [my neurologist is the ideal person to discuss exercise with]. I think [my neurologist] is a very intelligent man, and he takes care of more than my brain. That's one reason why I finally found him and I'm very glad. Because he's my third neurologist here in Illinois. Because I did not like the first two. Participant 42, SP, M, ≤ 54 years of age, insufficiently active, TSD>3 years.

Participants indicated that the source would have adjunct knowledge on exercise promotion, and this could be through the delivery of information via the formats discussed in Theme 1. Some individuals expressed that the exercise promotion could come initially from neurologists, and then be followed up with a referral to a physical therapist or a personal trainer. Many participants acknowledged that a source which had a combination of MS knowledge and exercise knowledge would be ideal.

I want one on one interaction initially… to address immediate issues that you're having, to get you back to your baseline. But I would also want to make sure that when you leave that you've got an action plan for you to maintain your health whether that be a home based system, …or they recommend that you work with a personal trainer once you leave if you can, if that's something that's feasible for you… Participant 19, Primary Progressive MS, F, ≤ 54 years of age, sufficiently active, TSD>3 years

There were participants who indicated a lack of confidence in a neurologist's ability to promote exercise. Some participants deemed that exercise was not within the neurologist's area of expertise and understood that this meant a different health‐care provider would promote exercise.

I'm not so sure it will be a neurologist (who I'd like information from). I would probably think it would be, maybe somebody like in kinesiology (exercise science) or a physical therapist with a sub‐specialty in my disease. Participant 19, Primary Progressive MS, F, ≤ 54 years of age, sufficiently active, TSD>3 years

#### 3.3.2 Exercise expertise

One‐third of participants expressed that the ideal source for exercise promotion would have professional understanding of exercise (eg education on exercise physiology). Few participants had experience in receiving health‐care interactions with professionals with exercise expertise; however, some participants envisioned that this would be a beneficial possibility for them. Participants felt such a source would have a good understanding of equipment, assessment and prescription of exercise. Participants deemed that such a source would understand exercise behaviour change (eg exercise goal setting and strategies to increase accountability) and that these areas would be prioritised when promoting exercise.

Someone that can say, “Okay, I know these are your weaknesses, but here is the activities that will help strengthen that or here is the thing that you can do to better that, or here is a plan to work up to 5 miles.” You know what I mean? Just giving more guidance. Participant 14, RR MS, F, >55 years of age, insufficiently active, TSD>3 years.

Individuals who emphasised a need for the source to be an expert in exercise primarily told us that physical therapists were the ideal source of exercise information. For some participants, this was because the physical therapists were the health‐care providers who had previously provided them with exercise information. Reasons for preferring physical therapists included a belief that physical therapists have increased knowledge of muscle physiology and knowledge of compensation strategies for physical disabilities.

…the physical therapist that specialize in MS people that … Oh, well, I guess from my experience with these two individuals, the physical therapist might know more about what I need to do to compensate for my physical deficits than the neurologist. Participant 9, SP MS, F, ≤ 54 years of age, sufficiently active, TSD≤3 years.

Some individuals mentioned personal trainers and other fitness professionals as the preferred profession to provide exercise promotion due to the desire for more detailed and specific exercise programmes and past experiences with personal trainers.

A physical trainer [would be the ideal source of exercise information], I know they know their way around the gym and they know it's like, if you're going to work on your core, here are the 5 or 6 exercises to work on. I don't know if there's ever been a physical trainer or therapist who said, okay here is the right routine for someone with MS.” “Because MS affects everyone differently. If someone had the problem was in their arms and their hands, okay here's what you should do. If you have problems with your legs here's what you should do. Participant 32, RR MS, M, ≤ 54 years of age, sufficiently active, TSD> 3 years

Participants who prioritised exercise knowledge from the exercise promotion source had a tendency to separate this need from a source with MS information. For example, those who deemed exercise to be a priority occasionally wanted that person to have a subspecialty in MS, or have professional experiencing with an MS population, but these traits were not required. Participants did deem it important that the source have experience working with clinical populations and persons with disability.

## 4 DISCUSSION

Exercise participation in the context of MS is a societal and clinical concern, as persons with MS are not engaging in sufficient exercise to accrue health benefits.^15^, ^16^ The current qualitative study indicated how patients with MS want to receive exercise promotion and identified the ideal source to receive this information. Persons with MS want to receive information on exercise promotion in multiple formats, and they want to receive exercise promotion from professionals primarily expert in MS. These data are the first to establish the preferred information format, and we now identify that health‐care expertise is important to patients when receiving exercise promotional information.

Research from patients indicates that advice from health‐care providers may be very effective in changing a patient's exercise behaviour,^45^ and there is strong evidence for exercise being one of the best therapies available for managing symptoms^12^, ^13^ and must be included in the comprehensive care of patients with MS.^12^ We recently established that patients with MS have a strong interest in receiving information on exercise promotion from health‐care providers, yet they might not be receiving it from through health‐care providers.^17^ We further established the needs and wants of patients with MS including (i) information and knowledge on the benefits of exercise and exercise prescription, (ii) materials to allow home and community exercise, and (iii) tools for initiating and maintaining exercise behaviour.^17^ Identifying the preferred method to deliver information on exercise promotion to persons with MS is important as we move closer to creating a conceptual model and toolbox for the promotion of exercise through the patient and health‐care provider interaction.

### 4.1 Information format

To ensure comprehensive exercise promotion, we must consider delivery of information and resources over three different formats. This was our first theme. We established that *the traditional in‐person patient‐provider clinical consultation, print media* and *electronic media* are all acceptable formats for persons with MS to receive exercise promotion. These information formats are recognised as common channels that individuals with MS choose when seeking health^46^ and physical activity^22^ information. For example, researchers have established that electronic media (ie the Internet) was the most first source chosen when persons with MS are seeking health information and that the person's doctor or health‐care providers are the most trusted source for health information.^44^ When seeking information on physical activity, the preferred sources of information were the Internet and health‐care providers (ie physicians and allied care professionals).

We identify new information as to the preferred format for exercise promotion information. Patients with MS had personal preferences for individual formats, and many wanted reinforcement and explanation of information in cumulative formats. For example, attending an in‐person clinical consultation with a health‐care provider could be supplemented with take‐home print media. Health‐care providers might consider identifying print media that encourages exercise promotion. Future research should develop new and improved exercise promotion print media for health‐care providers to deliver among patients with MS. Participants indicated that information provided in electronic format (ie websites and email) may provide difficulty for them and acknowledged that this was because of a lack of confidence with technology or being overwhelmed with online information sources. These results are similar to problems identified by persons with MS when seeking information online, as 40% of persons with MS are concerned about the quality of online health information, and 21% of persons with MS have indicated online information is difficult to understand.^44^ Health‐care providers might consider their role in disseminating electronic information as one which directs patients to credible online sources.^22^ Health‐care providers might have an important role in directing patients to trusted and reliable electronic media for exercise promotion in MS, and this evidence indicates a need to research and develop relevant and easily understandable electronic media on exercise promotion in MS.

### 4.2 Information source

Health‐care providers were considered trusted and credible sources of exercise information, and this is commonly perceived among person with MS.^44^, ^46^ We provide brand new information as to which professional expertise persons with MS deem important when it comes to exercise promotion. Professionals who are primarily experts in MS are deemed important messengers of exercise information, and this is consistent with previous research.^19^, ^22^ This is important as research indicates neurologists are the most frequently visited health‐care providers by persons with MS,^17^ and this profession might consider their importance in exercise promotion. Persons with MS further consider expertise in exercise as highly important, and this includes health‐care providers (eg physical therapists) as well as exercise professionals (eg exercise physiologists). These professions must consider their importance in the overall promotion of exercise in persons with MS. Notably, some participants highlight the initial role neurologists might have in initiating exercise behaviours and the importance of then liaising with those more knowledgeable in exercise.

Clinical implementation might involve coordination, liaison and improved communication between health‐care providers (eg neurologists and physical therapists), and this might be performed through traditional face‐to‐face meetings or through the use of modern technology to communicate clinical results and expert opinion. We acknowledged that many of our results were experiential in that participants needed and wanted health‐care promotion in formats and from sources that were familiar to them. Suggestions in the literature indicate that poor familiarity with health‐care information might reduce patients’ ability to access health‐care information.^47^ Therefore, we might better inform people with MS of the current exercise promotion options available to them and then seek to improve upon these models.

We note some limitations of this study, and these are countered by much strength. We acknowledge that we recruited persons with mild‐to‐moderate MS disability, and our results may not be applicable among those with severe disability. We recruited only persons from the Midwest USA, and patient experiences and access to health‐care services may differ across local and international borders, and it is therefore important for future investigation of patients’ preferred format and source of exercise information to be investigated globally. Our use of spiral analysis, involvement of multiple researchers to independently and jointly analyse data and triangulation of data sources are strengths of this study which help ensure the data are a true representation of what was said by participants. Our use of a participatory framework and adoption of patient involvement and engagement in the design, conduct and interpretation of our results lessen the impact of any potential researcher bias resulting from our belief that exercise is beneficial for persons with MS and that there is a need to increase overall participation by persons with MS. Further, our use of PPIE throughout the research process strengthens the importance of our findings.

## 5 CONCLUSION

The low level of exercise uptake in persons with MS is a societal and clinical concern. Recent research indicates that persons with MS want to receive information about exercise and its promotion from health‐care providers. The current data underscore how to provide exercise information to patients with MS and identify that many health‐care providers must be involved in exercise promotion. Based on the views and opinions of participants in our study, it is clear that we must ensure that health‐care providers are prepared to provide exercise information to patients, research and develop exercise promotion material in print media, and establish credible electronic sources of exercise promotion for persons with MS.
